# Differential HLA Association of GAD65 and IA2 Autoantibodies in North Indian Type 1 Diabetes Patients

**DOI:** 10.1155/2021/4012893

**Published:** 2021-12-27

**Authors:** Neihenuo Chuzho, Neeraj Kumar, Neetu Mishra, Nikhil Tandon, Uma Kanga, Gurvinder Kaur, Paras Singh, Gunja Mishra, Shreya Sharma, Narinder K. Mehra

**Affiliations:** ^1^Indian Council of Medical Research (ICMR)-National Institute of Pathology, Safdarjung Hospital Campus, New Delhi, India; ^2^Symbiosis School of Biological Sciences, Symbiosis International (Deemed University), Pune, India; ^3^Department of Endocrinology and Metabolism, All India Institute of Medical Sciences, New Delhi, India; ^4^Department of Transplant Immunology and Immunogenetics, All India Institute of Medical Sciences, New Delhi, India; ^5^Laboratory Oncology Unit, Dr BR Ambedkar Institute Rotary Cancer Hospital, All India Institute of Medical Sciences, New Delhi, India; ^6^Department of Molecular Medicine, National Institute of Tuberculosis and Respiratory Diseases, Sri Aurobindo Marg, New Delhi, India; ^7^Emeritus Scientist (ICMR), and Former Dean (Research), All India Institute of Medical Sciences, New Delhi, India

## Abstract

The human leucocyte antigen (HLA) association with type 1 diabetes (T1D) is well known but there are limited studies investigating the association between *β*-cell autoantibodies and HLA genes. We evaluated the prevalence of GAD65 and IA-2 autoantibodies (GADA and IA2A) in 252 T1D patients from North India and investigated the genetic association of GADA and IA2A with HLA class I and class II genes/haplotypes. GADA and IA2A were detected in 50.79% and 15.87% of T1D patients, respectively, while only 8.73% had both GADA and IA2A. HLA-DRB1^∗^03 was observed to be significantly higher in GADA+ T1D patients as compared to GADA– (91.41% vs. 66.13%, Bonferroni-corrected *P* (*P*_c_) = 1.11 × 10^−5^; OR = 5.45; 95% CI: 2.67-11.08). Similarly, HLA-DQB1^∗^02 was found to be significantly increased in GADA+ patients (94.53%, *P*_c_ = 2.19 × 10^−5^; OR = 6.27; 95% CI: 2.7-14.49) as compared to GADA– (73.39%). The frequencies of HLA-DRB1^∗^04 and DQB1^∗^03 were increased in IA2A+ patients (45.0% and 52.5%, respectively) as compared to that in IA2A– (25.94% and 33.96%, respectively). Further, the frequency of DRB1^∗^03-DQB1^∗^02 haplotype was found to be significantly increased in GADA+ T1D patients as compared to GADA- (60.55% vs. 41.94%, *P* = 3.94 × 10^−5^; OR = 2.13; 95%CI = 1.49-3.03). Similarly, HLA-DRB1^∗^04-DQB1^∗^03 haplotype was found to be significantly increased in IA2A+ T1D patients compared to IA2A– patients (22.5% vs. 12.97%; *P* = 0.041; OR = 1.95; 95%CI = 1.08-3.52). None of the HLA class I genes (HLA-A, B, and Cw) was found to be associated with GADA or IA2A in people with T1D. Our findings suggest that HLA-DRB1^∗^03/DQB1^∗^02 and HLA-DRB1^∗^04/DQB1^∗^03 might play an important role in the development of GADA and IA2A, respectively.

## 1. Introduction

Type 1 diabetes (T1D) is a multifaceted autoimmune disorder, which is caused by immune-mediated destruction of insulin producing islet *β*-cells and is characterised by insulin deficiency and the presence of islet autoantibodies in the peripheral blood [1, 2]. T1D is thought to be a result of complex interaction of environmental and genetic factors. Genetically, T1D is strongly associated with Human Leukocyte Antigen (HLA) genes present on the humans' chromosome 6p21 [3, 4]. A large number of family-based association and case-control studies on diverse population groups have reported independent or joint association of few common alleles at the HLA class I (A, B, and Cw) and HLA class II (DRB1, DQA1, and DQB1) loci [5–7]. Globally, HLA-DR3 (DRB1^∗^03:01-DQA1^∗^05:01-DQB1^∗^02:01) and some DR4 (DRB1^∗^04:01/05-DQA1^∗^03:01-DQB1^∗^03:02) haplotypes are reported to be the primary T1D predisposing haplotypes. On the contrary, HLA-DR2 (DRB1^∗^15:01-DQA1^∗^01:02-DQB1^∗^06:02) haplotype was reported to be negatively associated with T1D [8–11]. Our previous studies on North Indian population have revealed a strong genetic association of HLA-DRB1^∗^03 allele and its haplotypes with T1D, while DRB1^∗^07, ^∗^11, ^∗^13, and ^∗^15 were found to be negatively associated [12, 13]. Further, HLA-A^∗^02, A^∗^26, B^∗^08, and B^∗^50 at the class I locus were also observed to be strongly associated with T1D but this association was suggested to be secondary because of linkage disequilibrium of these alleles with the HLA-DRB1^∗^03 gene [13, 14].

HLA class II molecules execute their function in two different ways: first, by controlling T cell development and their autoimmune potential through interaction of HLA-peptide complex with T-cell receptor in the thymus [15–17] and second, by regulating presentation of antigens during inflammation [18]. Mostly, HLA-DQ molecule is regarded to be the key player in inducing the autoimmune response. For instance, among high-risk T1D haplotypes, DQB1^∗^03:02 has the major susceptibility effect and the protection is reported to be contributed by DQB1^∗^06:02 allele on the protective DR15 haplotypes [12]. Although the genetic associations are well-studied, there is limited studies detailing the association between the presence of a particular gene/allele and *β*-cell autoantibodies.

There are several *β*-cell-derived autoantigens identified as targets of T-cell's response as well as autoantibodies in people with T1D [17, 18]. Some of the important autoantibodies associated with *β*-cell autoimmunity are antibodies to 65 kDa form of Glutamic acid decarboxylase (GADA), tyrosine phosphatase-related islet antigen 2 (IA2A), insulin (IAA), and zinc transporter 8 (ZnT8A) [18, 19]. Studies have revealed that these autoantibodies are associated with the autoimmune processes which lead to T1D and the presence of two or more islet autoantibodies in peripheral blood is used as key biomarkers to identify individuals at risk of developing T1D and for disease diagnosis [2, 20, 21]. Previously, some studies using a small number of T1D patients revealed the most important autoantibodies to be GADA and IA2A in India [22–24]. Additional studies on recent-onset T1D patients and prediabetic subjects from other populations also reported GADA and/or IA2A to be developed at an early stage [25–28]. HLA genotypes have been reported to be differentially associated with islet autoantibodies such as GADA, IA2A, and IAA [29–31]. Krischer et al. in the TEDDY study (birth cohort of children at high-risk for T1D at the time of enrolment) reported the presence of GADA and IAA in 37.7% and 43.7%, respectively, in genetically susceptible children, GADA being more common in those with HLA-DR3/3 genotypes as compared to IAA. This study also reported that IAA was more common in HLA-DR4/DQ8 children, suggesting the important role of HLA-DR/DQ genes for the development of these autoantibodies [30]. In addition to genetic factors, the appearance of autoantibodies against *β*-cell autoantigens correlates with age and disease duration, indicating heterogeneity in the initiation of *β*-cell autoimmunity [32, 33]. It has been observed that such studies were carried out mostly in Caucasian population and there is lack of knowledge about the same in non-Caucasian groups. Our earlier studies on immunogenetic association of T1D from North India revealed a very strong association of only HLA-DR3-DQ2 haplotype with T1D and not of HLA-DR4-DQ8 [12, 13]. Furthermore, so far, to the best of our knowledge, no study has been conducted to evaluate the genetic association of HLA class I and class II genes/haplotypes with the presence of autoantibodies, particularly GADA and IA2A, in T1D patients from North India. Thus, in this study, we investigated the prevalence of GADA and IA2A in T1D patients from North India and their genetic association with HLA class I and class II genes/haplotypes.

## 2. Materials and Methods

### 2.1. Study Participants

Two hundred fifty-two T1D patients from North India were recruited during a period of three years (2016-2019) from Department of Endocrinology and Metabolism, All India Institute of Medical Sciences (AIIMS), New Delhi, India. The study was approved by the Ethics Committee of AIIMS. From each study participant, a written informed consent was obtained prior to the recruitment (consent of legal guardian was taken if the patient was a minor). The patients, who were willing to participate and provided the written informed signed consent, were included in the study. All the patients were clinically diagnosed as per the definition given in the Registry of Youth Onset Diabetes in India (YDR) [34] and had the age at disease onset equal or less than 35 years. The additional clinical details used to categorise these individuals as T1D are as follows: (i) the onset was usually acute and required insulin for survival, (ii) family history of diabetes was rare, (iii) presence of features of other associated autoimmune disorders and absence of obesity and acanthosis nigricans, and (iv) urine ketone positivity.

All the medical details of patients were obtained through a standardized patient history questionnaire, and the people without T1D or having other types of diabetes (e.g., T2D, GDM, and LADA) were excluded from the study. All the study subjects belonged to the northern regions of India, i.e., the union territory of Delhi, Punjab, Rajasthan, Uttar Pradesh, and Haryana. Approximately 5 ml blood was collected by venipuncture from each study participant; out of that, 2 ml was used for serum separation and the remaining 3 ml was used for the extraction of DNA using chloroform-ammonium acetate method as reported previously [35]. The DNA was dissolved in Tris-EDTA buffer, and its quality and quantity were assessed by a spectrophotometer.

### 2.2. Autoantibody Testing

Autoantibodies against GAD65 (GADA) and IA-2 (IA2A) in the sera of people with T1D were quantified using commercially available ELISA kits, namely, ElisaRSR™ GADAb kit and ElisaRSR™ IA-2 Ab ELISA Version 2 kit (RSR Ltd., Cardiff, United Kingdom). GADA and IA2A detection assays were performed as per the protocol provided by the kits' manufacturer. For GADA assay, based on the recommended assay cut-off value, the study subjects were defined as GAD65 autoantibody positive (GADA+, ≥5 U/ml) or negative (GADA–, <5 U/ml). Similarly, those with an IA-2 autoantibody titre of ≥7.5 U/ml were defined as IA2A positive (IA2A+) and those with IA2A titre < 7.5 U/ml were defined as IA2A negative (IA2A–). The reported specificity and sensitivity of RSR GADAb ELISA kit in the IASP 2013 study were 98% and 78%, respectively, whereas, the RSR IA-2 Ab ELISA kit achieved 98% specificity and 76% sensitivity in the IASP 2016 study. Further, the GADA kit has an interassay Coefficient of Variability (CV) of 5.7% and an intra-assay CV of 6.4% while the IA2A kit has an interassay CV of 4.3% and an intra-assay CV of 2.0%.

### 2.3. HLA Class I and Class II Genotyping

Low-resolution genotyping of HLA class I (A, B, and Cw) and class II (DRB1 and DQB1) was carried out using PCR- (Polymerase Chain Reaction-) SSP (sequence-specific primer) kits from Inno-Train Diagnostik (Kronberg, Germany). The PCR-SSP kits contained prealiquoted sequence-specific primers in a multiwelled plate. The composition of master mix and amplification parameters for PCR-SSP were followed as per the protocol provided by the manufacturer. The amplified products were run on 2% agarose gel, and the HLA genotype was determined according to the allele interpretation table supplied with the kit.

### 2.4. Statistical Analysis

The frequencies for all the HLA class I and II genes were calculated by directly counting the individuals positive for a specific allele and dividing them by total number of T1D patients. The haplotype frequencies were calculated by dividing the number of a particular haplotype by the total number of haplotypes (2N). The genetic association of HLA genes was analysed by comparing the frequency distribution among the study groups using chi-square test, and a *P* value < 0.05 was considered to be statistically significant. GraphPad Prism 5.0 Software (San Diego, California) was used to analyse the data, and the risk conferred by a specific HLA gene/haplotype was measured by estimating odds ratios (ORs) with 95% confidence interval (CI).

## 3. Results

### 3.1. Prevalence of GADA and IA2A and Effect of Age at Onset and Disease Duration on Autoantibody Status

Out of 252 T1D patients tested for the presence of autoantibodies against GAD65 (GADA) and tyrosine phosphatase-related islet antigen 2 (IA2A), GADA was present in 128 (50.79%) patients while IA2A was detected in 40 patients (15.87%) (Table 1). Further, both GADA and IA2A were detected in the sera of only 8.73% of the study subjects while 57.94% of patients had either GADA or IA2A. We found no difference in the age at sampling between GADA+ patients and GADA– patients. However, the median of age at sampling was lower in IA2A+ T1D patients (18 years) as compared to that in IA2A– patients (21 years) but the difference was not statistically significant.

Analysis of sex-wise distribution revealed a slightly higher number of males with T1D (52.78%) as compared to females (47.22%). Similarly, higher but not statistically significant numbers of males (53.12%) were found to have GADA as compared to females (46.88%). Interestingly, we found a significantly higher number (25 out of 40) of IA2A+ patients to be males (62.5% vs. 37.5% females, *P* = 0.025) (Table 1). When the age at onset and the disease duration of all the patients were analysed, the medians of age at onset and disease duration were observed to be comparable (11.5-12 years and 5.5-6 years, respectively) for all four groups of patients, i.e., GADA+, GADA–, IA2A+, and IA2A– (Table 1).

### 3.2. Association between HLA Class II Alleles and T1D Autoantibodies GADA and IA2A

HLA class II genes, namely, DRB1 and DQB1, were studied in 252 T1D patients to evaluate their association with the status of GADA and IA2A autoantibodies. HLA-DRB1^∗^03 allele was found to be significantly increased in T1D patients carrying GADA as compared to those without GADA (91.41% vs. 66.13, *P*_c_ = 1.11 × 10^−5^; OR = 5.45; 95% CI: 2.67-11.08). In contrast, we found HLA-DRB1^∗^15 to be significantly decreased in the GADA+ patients in comparison to the GADA– patients (11.72% vs. 22.58%, *P* = 0.034; OR = 0.45; 95% CI: 0.23-0.89), but the statistical significance of this difference was lost after Bonferroni correction (Bonferroni-corrected *P*, *P*_c_ = 0.442) (Table 2).

Further, HLA-DRB1^∗^04 allele was observed to be significantly increased in IA2A+ T1D patients as compared to IA2A– patients (45.0% vs. 25.94%, *P* = 0.025; OR = 2.34; 95% CI: 1.17-4.64), but after Bonferroni correction, the statistical significance of this difference was lost (*P*_c_ = 0.325). Among the remaining DRB1 alleles, HLA-DRB1^∗^15 showed a nonsignificant decrease in IA2A+ cases as compared to that in IA2A– (10.0% vs. 18.4%) (Table 2).

When we analysed the genotypic distribution of HLA-DRB1 genes in GADA+, GADA–, IA2A+, and IA2A– people with T1D (Table 2), we observed a significant increase in the frequency of DRB1^∗^03/^∗^03 homozygote in GADA+ vs. GADA– patients (29.69% vs. 17.74%; *P* = 0.038; OR = 1.96; 95% CI: 1.08-3.54); however, the statistical significance was lost after Bonferroni correction (*P*_c_ = 0.228). In contrast, DRB1^∗^XX/XX (where X is other than DRB1^∗^03 and ^∗^04 alleles) was found to be significantly decreased in GADA+ T1D patients as compared to that in the GADA– (3.91% vs. 28.23%; *P*_c_ = 7.68 × 10^−7^; OR = 0.13; 95% CI: 0.04-0.27). Among IA2A+ T1D patients, the frequency of DRB1^∗^03/^∗^04 heterozygote was found to be significantly increased (37.5%, *P* = 0.044; OR = 2.23; 95% CI: 1.09-4.54) as compared to that in IA2A– patients (21.23%) but after Bonferroni correction, the statistical significance of this difference was lost (*P*_c_ = 0.264). None of the study subjects with T1D was found to be HLA-DRB1^∗^04/^∗^04 homozygous.

The analysis of HLA-DQB1 genes with respect to GADA positivity in people with T1D (Table 3) revealed a significantly increased frequency of HLA-DQB1^∗^02 allele in GADA+ patients as compared to that in GADA– (94.53% vs. 73.39%, *P*_c_ = 2.19 × 10^−5^, OR = 6.27; 95% CI: 2.7-14.49). On the other hand, significantly lower frequencies of HLA-DQB1^∗^05 and DQB1^∗^06 alleles were observed in patients carrying GADA as compared to those in GADA– patients (7.81% vs. 28.23%, *P*_c_ = 1.17 × 10^−4^, OR = 0.21, 95% CI: 0.1-0.45, and 11.71% vs. 25.81%, *P*_c_ = 0.035, OR = 0.38, 95% CI: 0.2-0.74, respectively). Further, HLA-DQB1^∗^03 allele was found to be significantly higher in IA2+ T1D patients as compared to that in IA2A– patients (52.5% vs. 33.96%; *P* = 0.04; OR = 2.15; 95% CI: 1.09-4.22), but this statistical significance was lost after Bonferroni correction (*P*_c_ = 0.20) (Table 3). DQB1^∗^05 and DQB1^∗^06 were observed to be nonsignificantly reduced in IA2A+ patients (12.5% and 15.0%, respectively) as compared to those in IA2A– patients (18.87% and 19.34%, respectively).

A comparative genotypic distribution of HLA-DQB1 in autoantibody-positive and autoantibody-negative T1D patients is also shown in Table 3. Among the GADA+ patients, DQB1^∗^02/^∗^02 homozygote was found to be significantly increased as compared to that in GADA– patients (35.16% vs. 22.58%; *P* = 0.028; OR = 1.86; 95% CI: 1.07-3.23), but this statistical significance was lost after Bonferroni correction (*P*_c_ = 0.168). Interestingly, only 1 (0.78%) out of 128 GADA+ patients was found to have DQB1^∗^XX/XX (where X is an allele other than DQB1^∗^02 or DQB1^∗^03) as compared to 19 (15.32%) out of 124 GADA– patients and this difference was found to be statistically significant (*P*_c_ = 1.18 × 10^−4^; OR = 0.04; 95% CI: 0.01-0.26).

Further, DQB1^∗^02/^∗^03 heterozygote was found to be significantly increased in IA2A+ patients (47.5%, *P* = 0.009, OR = 2.65; 95% CI: 1.33-5.26) as compared to that in the IA2A– patients (25.47%), but after Bonferroni correction, this statistical significance was lost (*P*_c_ = 0.054). In contrast, DQB1^∗^02/XX was observed to be significantly decreased in IA2A+ patients (10%; *P* = 0.019; OR = 0.27; 95% CI: 0.1-0.76) as compared to that in IA2A– patients (25.47%) but after Bonferroni correction, the difference was not observed to be statistically significant (*P*_c_ = 0.114) (Table 3).

### 3.3. Association of DRB1-DQB1 Haplotypes with GADA and IA2A

HLA-DRB1-DQB1 haplotypes were compared between GADA+ and GADA– as well as IA2A+ and IA2A– T1D patients (Table 4). We found significantly increased frequency of DRB1^∗^03-DQB1^∗^02 haplotype in GADA+ T1D patients as compared to GADA– (60.55% vs. 41.94%, *P* = 3.94 × 10^−5^; OR = 2.13; 95% CI =1.49-3.03). On the other hand, haplotypes, viz., DRB1^∗^15-DQB1^∗^06 and DRB1^∗^11-DQB1^∗^03, were observed to be significantly decreased in GADA+ T1D patients compared to those without GADA (4.3% vs. 9.68%, *P* = 0.028, OR = 0.42, 95%CI = 0.20-0.86 and 0.39% vs. 3.23%, *P* = 0.039, OR = 0.12, 95% CI = 0.02-0.73, respectively).

When we analysed the association of haplotypes of HLA-DRB1-DQB1 loci with IA2A positivity, we observed a significant increase in the frequency of DRB1^∗^04-DQB1^∗^03 in IA2A+ T1D patients compared to IA2A– patients (22.5% vs. 12.97%; *P* = 0.041; OR = 1.95; 95%CI = 1.08-3.52).

### 3.4. Association of HLA Class I Alleles with GADA and IA2A in T1D Patients

HLA class I (HLA-A, HLA-B, and HLA-Cw) typing was performed for 157 T1D patients to assess the correlation of HLA class I alleles with the status of GADA and IA2A. Out of all the HLA-A alleles analysed, HLA-A^∗^03, A^∗^24, A^∗^26, and A^∗^68 showed notable differences in their distribution frequencies between GADA+ and GADA– patients (23.08% vs. 13.92%, 29.49% vs. 36.71%, 19.23% vs. 26.58%, and 12.82% vs. 6.33%, respectively). On the other hand, the frequency of HLA-A^∗^02 and HLA-A^∗^26 alleles was found to be higher in IA2A+ T1D patients as compared to IA2A– patients (42.31% vs. 37.4% and 26.92% vs. 22.14%, respectively), while HLA-A^∗^11 and A^∗^33 were observed with reduced frequencies in IA2A+ patients as compared to those in the IA2A– patients (11.54% vs. 20.61% and 11.54% vs. 19.85%, respectively). However, none of these differences was statistically significant (Supplementary Table S1).

When we analysed HLA-B alleles, 6 out of 78 (7.69%) GADA+ patients carried HLA-B^∗^07 as compared to that in only 2 out of 79 (2.53%) GADA– patients. Similarly, increased frequency of HLA-B^∗^13 was observed in GADA+ patients as compared to that in GADA– (12.82% vs. 7.59%). In contrast, HLA-B^∗^15 was found to be lower in GADA+ patients in comparison to that in GADA– (7.69% vs. 15.2%). Further, HLA-B^∗^15 was observed to be reduced and B^∗^37 was increased in IA2A+ patients as compared to those in IA2A– (3.85% vs. 12.98% and 7.69% vs. 1.53%, respectively). However, all these differences were not statistically significant (Supplementary Table S2).

While HLA-Cw^∗^06 was found to be higher (35.9%), HLA-Cw^∗^12 was observed to be reduced (12.82%) in GADA+ patients as compared to that in the GADA– (27.85% and 20.25%, respectively) but these differences were not statistically significant (Supplementary Table S3). Out of all the HLA-Cw alleles, only Cw^∗^07 showed a marginal but nonsignificant increase in the IA2A+ patients (65.38%) as compared to that in the IA2A– (52.67%).

### 3.5. Association of HLA-A-B-Cw-DRB1-DQB1 Haplotypes with GADA and IA2A Autoantibodies

**Figure 1:**
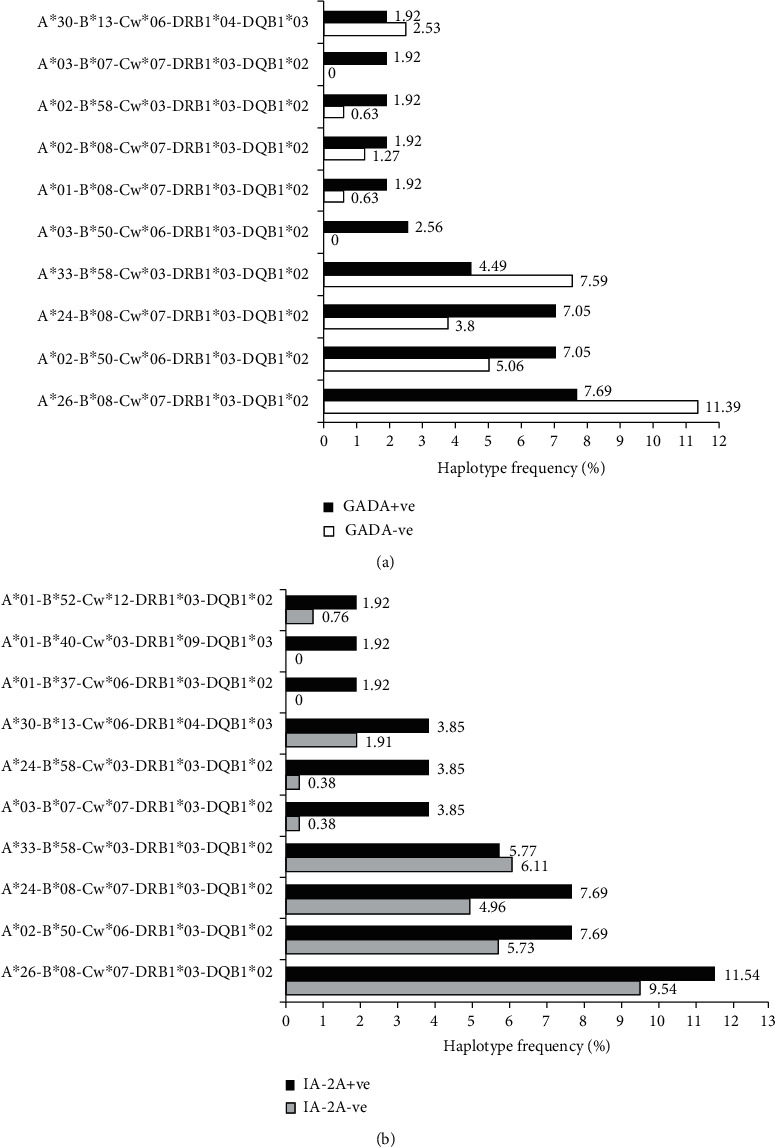
Comparative distribution of 10 most common HLA-A-B-Cw-DRB1-DQB1 haplotypes between (a) GADA+ (*N* = 78) and GADA– (*N* = 79) and (b) IA2A+ (*N* = 26) and IA2A– (*N* = 131) T1D patients from North India.

We studied the extended HLA haplotypes (A-B-Cw-DRB1-DQB1) and their association with GADA and IA2A status in 157 people with T1D. The top ten HLA-A-B-Cw-DRB1-DQB1 haplotypes that occurred most frequently in our study population are shown in Figure 1. While HLA-A^∗^26-B^∗^08-Cw^∗^07-DRB1^∗^03-DQB1^∗^02 was observed to be reduced in GADA+ cases (7.69%) as compared to that in the GADA– (11.39%), it was increased in IA2A+ cases (11.54%) as compared to IA2A– (9.54%). Further, HLA-A^∗^02-B^∗^50-Cw^∗^06-DRB1^∗^03-DQB1^∗^02 and HLA-A^∗^24-B^∗^08-Cw^∗^07-DRB1^∗^03-DQB1^∗^02 were observed to be increased in GADA+ (7.05% for both) and IA2A+ cases (7.69% for both) as compared to those in GADA– (5.06% and 3.8%, respectively) and IA2A– cases (5.73% and 4.96%, respectively). In contrast, HLA-A^∗^33-B^∗^58-Cw^∗^03-DRB1^∗^03-DQB1^∗^02 was observed to be reduced in GADA+ (4.49%) and IA2A+ cases (5.77%) as compared to that in the GADA– (7.59%) and IA2A– (6.11%) (Figure 1). We did not find these differences to be statistically significant.

## 4. Discussion

Our study revealed that GAD65 autoantibody (GADA) is present in one out of every two North Indian T1D patients while IA2A is comparatively lower (1 out of ~6 T1D patients). In the current study on North Indian type 1 diabetes patients, the prevalence of GADA and IA2A is found to be lower as compared to that in other populations of the world [28, 36, 37]. While the reason for the lower prevalence of GADA and IA2A is not known, it is in accordance with the earlier reports showing the lower autoantibodies in Indian T1D patients [38, 39]. We investigated whether the lower prevalence of GADA and IA2A is due to the effect of the age at disease onset and/or disease duration. We found that the median age at disease onset was comparable between autoantibody positive and autoantibody negative patients (i.e., GADA+ versus GADA– and IA2A+ versus IA2A–), which suggests that the two groups (autoantibody positive and autoantibody negative) do not differ as far as age at disease onset is concerned. Therefore, the two groups of our study population are comparable for the comparative analysis of HLA distribution. With regard to the age of the patient at sampling, we did not find any difference between GADA+ and GADA– patients but the age at sampling was nonsignificantly lower in patients with IA2A compared to those without IA2A. Autoantibodies are known to decline with disease duration [28, 38], and longer disease duration might have an impact on the presence of autoantibodies. In our study, the median disease duration is six years; therefore, our study population may not be representative to the newly diagnosed T1D patients. Further, we could not observe a correlation between disease duration and presence of GADA and IA2A suggesting that these autoantibodies may persist in the patients' sera long after the onset of the disease. However, the lack of correlation of age at disease onset and disease duration with the presence of GADA and IA2A may be because of a limited number of study participants; hence, a study with much larger sample size including both recent and long-standing T1D patients is desirable to draw definite conclusions about the correlation of age at disease onset and disease duration with the presence of autoantibodies against *β*-cell antigens.

Further, not only the proportion of male T1D patients was higher than female patients; the presence of GADA was also comparatively higher in male patients. This is in contrast to other populations like Europeans and Chinese who have a lower percentage of males with T1D having GADA as compared to females [28, 37, 40, 41]. The prevalence of IA2A has been reported to be comparable or slightly lower in male with T1D as compared to females [28, 37, 40]. However, in our study, IA2A was observed to be significantly higher in male as compared to females with T1D.

Our study focused only on two commonly used serological biomarkers for T1D, GADA and IA2A, and so, one of the limitations of this study is that we have not included other autoantibodies like as ZnT8A and IAA. Insulin autoantibody (IAA) was excluded as almost all the study participants were exposed to exogenous insulin at the time of sampling and insulin exposure could induce high titre of insulin autoantibodies in T1D patients [42]. Further, ZnT8A and islet cell autoantibodies such as ICA-12 were not studied because of resource limitations. Earlier studies on a smaller number of patients have reported a high prevalence of ICA-12 autoantibodies in Indians but ICA-12 autoantibody was reported to be nonspecific [33, 43, 44]. Other studies have also reported the similar prevalence of ZnT8A and IA2A [45–47] but these studies have not explored the correlation between autoantibody status and HLA genes/haplotype. Although an earlier study has reported that ZnT8A may be a better serological marker than IA2A for T1D in India [48], however, they reported their findings in a very small group of patients (88 T1D patients) and hence, more studies need to be done in this regard. Therefore, noninclusion of ZnT8A in our study is a major limitation. Earlier studies have shown that with the presence of just one type of autoantibody, there is a low risk of progression to clinical onset, whereas the appearance of two or more autoantibodies increases the risk [2, 21]. In our study, we found that only 8.73% of T1D patients had both GADA and IA2A which is quite low and implies the need for screening the presence of more types of autoantibodies in this population for risk assessment and disease diagnosis. The low prevalence of IA2A in our study population also indicates that along with GADA, additional serological markers such as ZnT8A and other autoantibodies against *β*-cell-associated autoantigens may also be explored to give more strength to autoantibodies as the diagnostic serological markers for T1D in Indian population. Further, as mentioned earlier, we collected the sample from each subject at a single time point and did not follow up to study participants for the persistence of the autoantibody positivity. Also, we did not look into the age of seroconversion as this was beyond our study objectives. Further studies are required to look at the seroconversion and persistence of the autoantibodies and how these associate with HLA genes/haplotypes.

HLA is known to be associated with T1D development, and the risk of disease can be ascribed to specific genotype or haplotype [49]. However, it is debatable whether HLA affects the generation of autoantibodies, the T1D development, or both [50, 51]. Our study showed that HLA-DRB1^∗^03 and HLA-DQB1^∗^02 alleles are strongly associated with the presence of GADA but not IA2A in T1D patients from North India. Further, in this population, HLA-DRB1^∗^04 and HLA-DQB1^∗^03 alleles were observed to be associated with IA2A. In our earlier immunogenetic association study, we found a significant association of six HLA class II haplotypes, namely, DRB1^∗^03-DQB1^∗^02, DRB1^∗^15-DQB1^∗^06, DRB1^∗^11-DQB1^∗^03, DRB1^∗^04-DQB1^∗^03, DRB1^∗^13-DQB1^∗^06 and DRB1^∗^07-DQB1^∗^02 with T1D [13] and hence, in this study, we also evaluated the association of these six haplotypes with the presence of GADA and IA2A. We found DRB1^∗^03-DQB1^∗^02 and DRB1^∗^04-DQB1^∗^03 to be significantly associated with the presence of GADA and IA2A, respectively. It is well recognised that both DRB1^∗^03 and DRB1^∗^04 are in strong linkage disequilibrium with DQB1^∗^02 and DQB1^∗^03, respectively. Therefore, it is difficult to say whether the associations of DRB1 and DQB1 are independent or because of the strong linkage disequilibrium between these two loci. Further, it remains unknown whether GADA and IA2A observed in T1D patients are directed to same antigenic GAD65 and IA2 peptides, respectively, or to diverse peptides. Since, different antigen peptides are presented by different HLA class II molecules to the CD4 T-cells, which then help B cells to generate antibodies against these antigenic peptides, it is easy to correlate autoantibody specificity in terms of antigen presentation by HLA class II molecules. Earlier studies have shown that most of the peptides derived from GAD65 and IA2 bind promiscuously to all class II molecules with a very few exceptions [52–54]. It is assumed that the allelic polymorphism, which influences the binding of specific peptide, is also responsible for the HLA association of T1D but the precise mechanism of association is not known. In the context of peptide binding, DR3 and DQ2 are highly selective but still, we observed a very strong association of DRB1^∗^03 and DQB1^∗^02 with GADA but not IA2A. Further, we observed a weak association of DRB1^∗^04 and DQB1^∗^03 with IA2A but not GADA. The lack of associations of IA2A with DRB1^∗^03/DQB1^∗^02 and GADA with DRB1^∗^04/DQB1^∗^03 suggests that IA2A and GADA could be generated through selective antigen presentation by the HLA molecules encoded by HLA-DRB1^∗^03/DQB1^∗^02 and DRB1^∗^04/DQB1^∗^03 genes, respectively. HLA class II molecules may participate in the presentation of different autoantigenic peptides to functionally diverse T cell subsets leading to the generation of diabetogenic T cell phenotypes. While predisposing allele may trigger a Th1 immune response, the protective alleles may prompt a Th2-type response [55, 56]. Another possible explanation for the observed associations of HLA DRB1^∗^03 and/or DQB1^∗^02 alleles with GADA is that because of the presentation of very limited GAD65 peptides, the DR3 and/or DQ2 molecule may be incapable of performing the thymic negative selection of GAD65-specific autoreactive T cell clones during the maturation of T cells, resulting in the escape of few GAD65-reactive T cell clones from the thymus to the periphery. This can lead to cellular as well as humoral autoimmune response against GAD65 in pancreas. Similar explanation may be given for the observed association of HLA-DRB1^∗^04 and DQB1^∗^03 with IA2A. However, this is just a hypothetical explanation and thus remains to be proved by functional studies.

Our data on class I genes, i.e., HLA-A, B. and Cw did not reveal significant association with GADA and IA2A suggesting no or minimal contribution of class I molecules in the generation of these autoantibodies. HLA class I and class II molecules present the foreign antigen or autoantigenic peptides to different T cell phenotypes, i.e., CD8 and CD4 T-cells, respectively, and there is a difference in the peptides presented by these molecules. This suggests that the manner in which certain HLA-A, B, Cw, DRB1, and DQB1 alleles occur on a specific extended HLA haplotype can have complementary and synergistic effects on multiple stages of autoimmune response. Our earlier studies have shown the strong association of A^∗^26-B^∗^08-DRB1^∗^03-DQB1^∗^02, A^∗^24-B^∗^08-DRB1^∗^03-DQB1^∗^02, A^∗^02-B^∗^50-DRB1^∗^03-DQB1^∗^02 and A^∗^33-B^∗^58-DRB1^∗^03-DQB1^∗^02 with T1D in Indian population [5, 13]. Although, all the HLA-DRB1^∗^03+ and DRB1^∗^04+ haplotypes were conserved on DQB1 locus (i.e., DRB1^∗^03-DQB1^∗^02 and DRB1^∗^04-DQB1^∗^03, respectively), they differed at HLA class I alleles. Therefore, we analysed the association between HLA-A-B-Cw-DRB1-DQB1 haplotypes and GADA and IA2A status of T1D patients. Interestingly, the top four haplotypes were found to be the same, viz., HLA-A^∗^26-B^∗^08-Cw^∗^07-DRB1^∗^03-DQB1^∗^02, A^∗^02-B^∗^50-Cw^∗^06-DRB1^∗^03-DQB1^∗^02, A^∗^24-B^∗^08-Cw^∗^07-DRB1^∗^03-DQB1^∗^02 and HLA-A^∗^33-B^∗^58-Cw^∗^03-DRB1^∗^03-DQB1^∗^02 in all the four groups of patients irrespective of their autoantibody status, i.e., GADA+, GADA–, IA2A+, and IA2A– suggesting their predominant role in the T1D development. Further, these haplotypes were also observed to differ in frequencies between autoantibody positive and negative patients but the differences were not statistically significant reiterating a minimal role of HLA-class I genes in GADA and IA2A autoantibody development. This may be because of the possibility that HLA class I antigen-mediated cytotoxic T lymphocyte (CTL) immune response may play a very important role in the destruction of islet *β*-cells resulting in the clinical manifestation of T1D but may not be directly involved in the autoantibody generation. This is also supported by other studies which revealed the association of class I alleles and extended haplotypes with the difference in the age at onset of T1D [57–59]. However, the precise mechanism by which certain HLA class I and class II molecules lead to faster and more severe autoimmune destruction of pancreatic islet *β*-cells is not clear. Another possibility of observing lack of association of class I alleles and their haplotypes with autoantibody status of the T1D patients could be because of the smaller number of study subjects, which is a major limitation of our study, and therefore, possibly, a larger number of patients should be studied in future in order to further determine the association between the T1D-associated haplotypes and autoantibody positivity.

## 5. Conclusion

To the best of our knowledge, this is the first time that the association between two most widely used T1D serological markers, GADA and IA2A, and HLA genes has been done in an Indian population. Under this study, the predominant autoantibody in Indian T1D patients is observed to be GADA while the occurrence of IA2A is quite low and hence, additional autoantibodies can also be looked for diagnosis of T1D in the Indian population. A major limitation of our study is that majority of the patients were long-term T1D patients and autoantibodies are known to decline with duration of the disease. Hence, lower prevalence of GADA and IA2A in T1D patients under this study could be because of longer disease duration and may not be representative to newly diagnosed T1D patients. Our study revealed that HLA-DRB1^∗^03, DQB1^∗^02 alleles, and their haplotypes strongly associate with the presence of autoantibodies against GAD65 while HLA-DRB1^∗^04, DQB1^∗^03 alleles, and their haplotypes are associated with autoantibodies against IA-2 in T1D patients. Further, HLA class I genes have no or minimal role in the development of GAD65 and IA-2 autoantibodies. Studying the immunogenetic and immunological markers of T1D along with the GADA and IA2A-mediated autoimmune response can help in understanding the mechanism that may ultimately lead to *β*-cell death. Therefore, it can pave the way for developing therapeutic approaches for halting the *β*-cell autoimmunity in its initial phase.

## Figures and Tables

**Table 1:** Demographic details of 252 type 1 diabetes patients from North India.

Phenotype	All T1D subjects	GADA		IA2A		GADA+ and IA2A+	Either GADA+ or IA2A+
		Positives	Negatives	Positives	Negatives		
*N* (%)	252	128 (50.79)	124 (49.21)	40 (15.87)	212 (84.13)	22 (8.73)	146 (57.94)

Age at onset (years)							
Median (range)	12 (6 m-35)	12 (6 m-35)	12 (3-31)	11.5 (3-32)	12 (6 m -35)	11 (6 m-25)	12 (6 m-35)

Age at sampling (years)							
Median (range)	21 (2-53)	21 (2-52)	20 (5-49)	18 (3-25)	21 (2-53)	20 (2-35)	21 (2-52)

Duration (years)							
Median (range)	6 (1 m-32)	6 (1 m-30)	6 (1-32)	5.5 (1 m-21)	6 (1-32)	5 (1 m-17)	6 (1 m-30)

Sex							
Male, *N* (%)	133 (52.78)	68 (53.12)	65 (52.42)	25 (62.5)	108 (50.94)	14 (63.6)	80 (54.8)
Female, *N* (%)	119 (47.22)	60 (46.88)	59 (47.58)	15 (37.5)	104 (49.06)	8 (36.4)	66 (45.2)

Abbreviations: *N*: total number of subjects; T1D: type 1 diabetes; m: month(s).

**Table 2:** Association of the HLA-DRB1 alleles and HLA-DRB1 genotypes with GADA and IA2A in 252 T1D patients.

		GADA					IA2A				
		Positives (%GF) (*N* = 128)	Negatives (%GF) (*N* = 124)	OR (95% CI)	*P* value	*P*_c_	Positives (%GF) (*N* = 40)	Negatives (%GF) (*N* = 212)	OR (95% CI)	*P* value	*P*_c_
DRB1 allele	^∗^01	1 (0.78)	2 (1.61)	0.48 (0.06-3.72)	0.543		0 (0)	3 (1.41)	0 (0-6.88)	1	
	^∗^03	117 (91.41)	82 (66.13)	5.45 (2.67-11.08)	*8.55 × 10^−7^*	*1.11 × 10^−5^*	32 (80.0)	167 (78.77)	1.08 (0.47-2.45)	1	
	^∗^04	41 (32.03)	32 (25.81)	1.35 (0.79-2.34)	0.342		18 (45.0)	55 (25.94)	2.34 (1.17-4.64)	*0.025*	0.325
	^∗^07	16 (12.5)	20 (16.13)	0.74 (0.37-1.5)	0.52		4 (10.0)	32 (15.09)	0.62 (0.22-1.8)	0.55	
	^∗^08	0 (0)	1 (0.81)	0 (0-3.73)	0.987		0 (0)	1 (0.47)	0 (0-20.65)	1	
	^∗^09	7 (5.47)	1 (0.81)	7.12 (1.12-44.84)	0.08		2 (5.0)	6 (2.83)	1.81 (0.4-8.197)	0.821	
	^∗^10	2 (1.56)	8 (6.45)	0.23 (0.05-0.98)	0.096		0 (0)	10 (4.72)	0 (0-1.98)	0.337	
	^∗^11	1 (0.78)	7 (5.65)	0.13 (0.02-0.84)	0.065		1 (2.5)	7 (3.3)	0.75 (0.12-4.87)	1	
	^∗^12	1 (0.78)	3 (2.42)	0.32 (0.045-2.26)	0.592		0 (0)	4 (1.89)	0 (0-5.14)	0.852	
	^∗^13	5 (3.91)	12 (9.68)	0.38 (0.135-1.07)	0.115		3 (7.5)	14 (6.6)	1.15 (0.34-3.93)	1	
	^∗^14	6 (4.69)	12 (9.68)	0.46 (0.17-1.22)	0.196		2 (5.0)	16 (7.55)	0.645 (0.16-2.64)	0.811	
	^∗^15	15 (11.72)	28 (22.58)	0.45 (0.23-0.895)	*0.034*	0.442	4 (10.0)	39 (18.4)	0.49 (0.17-1.41)	0.29	
	^∗^16	1 (0.78)	4 (3.23)	0.24 (9.03-1.60)	0.348		2 (5.0)	3 (1.41)	3.67 (0.71-19.07)	0.383	

DRB1/DRB1 genotype	03/03	38 (29.69)	22 (17.74)	1.96 (1.08-3.54)	*0.038*	0.228	7 (17.5)	53 (25)	0.64 (0.27-1.5)	0.413	
	03/04	35 (27.34)	25 (20.16)	1.49 (0.83-2.68)	0.234		15 (37.5)	45 (21.23)	2.23 (1.09-4.54)	*0.044*	0.264
	03/XX	44 (34.37)	35 (28.23)	1.33 (0.78-2.27)	0.360		10 (25)	69 (32.55)	0.69 (0.32-1.48)	0.448	
	04/04	0 (0)	0 (0)	NA	1		0 (0)	0 (0)	NA	1	
	04/XX	6 (4.69)	7 (5.65)	0.82 (0.28-2.41)	0.953		3 (7.5)	10 (4.72)	1.64 (0.46-5.83)	0.734	
	XX/XX	5 (3.91)	35 (28.23)	0.13 (0.04-0.27)	*1.28 × 10^−7^*	*7.68 × 10^−7^*	5 (12.5)	35 (16.51)	0.72 (0.27-1.92)	0.689	

Abbreviations: GF: gene/genotype frequency; CI: confidence interval; OR: odds ratio; *P*_c_: Bonferroni-corrected *P* value; NA: not available; X: an allele other than DRB1^∗^03 and ^∗^04. Statistically significant values are given in italic.

**Table 3:** Association of the HLA-DQB1 alleles and HLA-DQB1 genotypes with GADA and IA2A in 252 T1D patients.

		GADA					IA2A				
		Positives (%GF) (*N* = 128)	Negatives (%GF) (*N* = 124)	OR (95% CI)	*P* value	*P*_c_	Positives (%GF) (*N* = 40)	Negatives (%GF) (*N* = 212)	OR (95% CI)	*P* value	*P*_c_
DQB1 allele	^∗^ *02*	*121 (94.53)*	*91 (73.39)*	*6.27 (2.7-14.49)*	*4.39 × 10^−6^*	*2.19 × 10^−5^*	33 (82.5)	179 (84.43)	0.87 (0.36-2.08)	0.943	
	^∗^03	48 (37.5)	45 (36.29)	1.05 (0.63-1.75)	0.945		21 (52.5)	72 (33.96)	2.15 (1.09-4.22)	*0.04*	0.20
	^∗^04	0 (0)	2 (1.61)	0 (0-1.86)	0.464		0 (0)	2 (0.94)	0 (0-10.35)	1	
	^∗^05	10 (7.81)	35 (28.23)	0.21 (0.1-0.45)	*2.34 × 10^−5^*	*1.17 × 10^−4^*	5 (12.5)	40 (18.87)	0.61 (0.23-1.62)	0.46	
	^∗^06	15 (11.71)	32 (25.81)	0.38 (0.2-0.74)	*0.007*	*0.035*	6 (15.0)	41 (19.34)	0.74 (0.3-1.83)	0.671	

DQB1/DQB1 genotype	02/02	45 (35.16)	28 (22.58)	1.86 (1.07-3.23)	*0.028*	0.168	10 (25)	63 (29.72)	0.79 (0.37-1.69)	0.679	
	02/03	42 (32.81)	31 (25)	1.47 (0.85-2.53)	0.219		19 (47.5)	54 (25.47)	2.65 (1.33-5.26)	*0.009*	0.054
	02/XX	34 (26.56)	32 (25.81)	1.04 (0.6-1.82)	1		4 (10)	62 (29.25)	0.27 (0.1-0.76)	*0.019*	0.114
	03/03	2 (1.56)	3 (2.42)	0.64 (0.13-3.27)	0.971		0 (0)	5 (2.36)	0 (0-4.09)	0.717	
	03/XX	4 (3.12)	11 (8.87)	0.33 (0.11-1.02)	0.097		2 (5)	13 (6.13)	0.81 (0.2-3.35)	1	
	XX/XX	1 (0.78)	19 (15.32)	0.04 (0.01-0.26)	*1.96 × 10^−5^*	*1.18 × 10^−4^*	5 (12.5)	15 (7.08)	1.88 (0.67-5.31)	0.398	

Abbreviations: GF: gene/genotype frequency; CI: confidence interval; OR: odds ratio; *P*_c_: Bonferroni-corrected *P* value; NA: not available; X: an allele other than DRB1^∗^03 and ^∗^04. Statistically significant values are given in italic.

**Table 4:** Comparative distribution of T1D-associated HLA-DRB1-DQB1 haplotypes and their association with the presence or absence of GADA and IA-2A in 252 T1D patients.

DRB1-DQB1 haplotype	GADA				IA2A			
	Positives (%HF) (2*N* = 256)	Negatives (%HF) (2*N* = 248)	OR (95% CI)	*P* value	Positives (%HF) (2*N* = 80)	Negatives (%HF) (2*N* = 424)	OR (95% CI)	*P* value
DRB1^∗^03-DQB1^∗^02	155 (60.55)	104 (41.94)	2.13 (1.49-3.03)	*3.94 × 10^−5^*	39 (48.75)	220 (51.89)	0.88 (0.55-1.42)	0.694
DRB1^∗^15-DQB1^∗^06	11 (4.30)	24 (9.68)	0.42 (0.20-0.86)	*0.028*	3 (3.75)	32 (7.55)	0.49 (0.16-1.56)	0.357
DRB1^∗^11-DQB1^∗^03	1 (0.39)	8 (3.23)	0.12 (0.02-0.73)	*0.039*	1 (1.25)	8 (1.89)	0.66 (0.11-4.13)	1
DRB1^∗^04-DQB1^∗^03	41 (16.02)	32 (12.90)	1.29 (0.78-2.11)	0.387	18 (22.5)	55 (12.97)	1.95 (1.08-3.52)	*0.041*
DRB1^∗^13-DQB1^∗^06	4 (1.56)	10 (4.03)	0.38 (0.12-1.16)	0.157	3 (3.75)	11 (2.59)	1.46 (0.43-5.00)	0.837
DRB1^∗^07-DQB1^∗^02	11 (4.30)	15 (6.05)	0.70 (0.32-1.52)	0.492	4 (5.0)	22 (5.19)	0.96 (0.34-2.75)	1

Abbreviations: 2*N*: diploid; HF: haplotype frequency; CI: confidence interval; OR: odds ratio. Significant values are italicized.

## Data Availability

Data is available on request.

## Supplementary Materials

Supplementary Materials Supplementary Table S1: comparative distribution of HLA-A alleles in 157 T1D patients with or without GADA and IA2A. Supplementary Table S2: comparative distribution of HLA-B alleles in 157 T1D patients with or without GADA and IA2A. Supplementary Table S3: comparative distribution of HLA-Cw alleles in 157 T1D patients with or without GADA and IA2A.
