# Supplementary material for: Differential HLA Association of GAD65 and IA2 Autoantibodies in North Indian Type 1 Diabetes Patients
Source: J Diabetes Res. 2021 Dec 27;2021:4012893. doi: 10.1155/2021/4012893 (PMC8723877; doi:10.1155/2021/4012893)
Supplement: Supplementary Materials — Supplementary Table S1: comparative distribution of HLA-A alleles in 157 T1D patients with or without GADA and IA2A. Supplementary Table S2: comparative distribution of HLA-B alleles in 157 T1D patients with or without GADA and IA2A. Supplementary Table S3: comparative distribution of HLA-Cw alleles in 157 T1D patients with or without GADA and IA2A. [file 4012893.f1.docx]

**SUPPLEMENTARY MATERIAL**

**Association of HLA class I alleles with GADA and IA2A in T1D Patients**

To evaluate the correlation of HLA class I alleles with the presence of GADA and IA2A, we performed HLA class I typing for 157 type 1 diabetes patients. The comparative distribution of HLA-A, -B and -Cw alleles with and without GADA and IA2A are shown in supplementary tables S1, S2 and S3 respectively. Our data revealed that none of the HLA-A, -B and Cw alleles showed significant difference in their distribution frequencies between GADA+ and GADA–as well as between IA2A+ and IA2A– type 1 diabetes patients in North India.

**Supplementary Table S1.** Comparative distribution of HLA-A alleles in 157 T1D patients with or without GADA and IA2A.

| **HLA-A Alleles** | **GADA** | | | | **IA2A** | | | |
| --- | --- | --- | --- | --- | --- | --- | --- | --- |
|  | **Positives (%GF)**  **(N=78)** | **Negatives (%GF)**  **(N=79)** | **OR (95% CI)** | **P-value** | **Positives (%GF)**  **(N=26)** | **Negatives (%GF)**  **(N=131)** | **OR (95% CI)** | **P-value** |
| *01 | 8 (10.25) | 8 (10.25) | 0.58 (0.23-1.46) | 0.365 | 3 (11.54) | 18 (13.74) | 0.82 (0.24-2.84) | 1 |
| *02 | 29 (37.18) | 29 (37.18) | 0.92 (0.48-1.74) | 0.919 | 11 (42.31) | 49 (37.4) | 1.23 (0.53-2.85) | 0.803 |
| *03 | 18 (23.08) | 18 (23.08) | 1.86 (0.82-4.18) | 0.203 | 5 (19.23) | 24 (18.32) | 1.06 (0.38-3.01) | 1 |
| *11 | 14 (17.95) | 14 (17.95) | 0.86 (0.39-1.89) | 0.87 | 3 (11.54) | 27 (20.61) | 0.50 (0.15-1.698) | 0.423 |
| *23 | 1 (1.28) | 1 (1.28) | 1.01 (0.10-9.87)) | 1 | 0 (0) | 2 (1.53) | 0.00 (0.00-9.92) | 1 |
| *24 | 23 (29.49) | 23 (29.49) | 0.72 (0.37-1.40) | 0.429 | 10 (38.46) | 42 (32.06) | 1.32 (0.56-3.12) | 0.685 |
| *25 | 1 (1.28) | 1 (1.28) | NA | 0.995 | 0 (0) | 1 (0.76) | 0.00 (0.00-19.78) | 1 |
| *26 | 15 (19.23) | 15 (19.23) | 0.66 (0.31-1.38) | 0.365 | 7 (26.92) | 29 (22.14) | 1.296 (0.51-3.32) | 0.783 |
| *29 | 0 (0) | 0 (0) | 0.0 (0.0-1.94) | 0.482 | 0 (0) | 2 (1.53) | 0.00 (0.00-9.92) | 1 |
| *30 | 7 (8.97) | 7 (8.97) | 1.46 (0.47-4.56) | 0.746 | 3 (11.54) | 9 (6.87) | 1.77 (0.48-6.58) | 0.679 |
| *31 | 1 (1.28) | 1 (1.28) | 0.50 (0.06-3.92) | 1 | 0 (0) | 3 (2.29) | 0.00 (0.00-6.58) | 1 |
| *32 | 3 (3.85) | 3 (3.85) | 3.12 (0.43-22.17) | 0.603 | 0 (0) | 4 (3.05) | 0 (0-4.899) | 0.825 |
| *33 | 13 (16.67) | 13 (16.67) | 0.79 (0.36-1.75) | 0.709 | 3 (11.54) | 26 (19.85) | 0.53 (0.16-1.78) | 0.471 |
| *43 | 1 (1.28) | 1 (1.28) | NA | 0.995 | 1 (3.85) | 0 (0) | NA | 0.367 |
| *68 | 10 (12.82) | 10 (12.82) | 2.18 (0.0.74-6.4) | 0.266 | 2 (7.69) | 13 (9.92) | 0.76 (0.18-3.23) | 1 |

Abbreviations: GF, gene frequency; CI, confidence interval; OR, odds ratio.

**Supplementary Table S2**. Comparative distribution of HLA-B alleles in 157 T1D patients with or without GADA and IA2A.

| **HLA-B Alleles** | **GADA** | | | | **IA2A** | | | |
| --- | --- | --- | --- | --- | --- | --- | --- | --- |
|  | **Positives (%GF)**  **(N=78)** | **Negatives (%GF)**  **(N=79)** | **OR (95% CI)** | **P-value** | **Positives (%GF)**  **(N=26)** | **Negatives (%GF)**  **(N=131)** | **OR (95% CI)** | **P-value** |
| *07 | 6 (7.69) | 2 (2.53) | 3.21 (0.71-14.31) | 0.268 | 2 (7.69) | 6 (4.58) | 1.74 (0.38-8.09) | 0.864 |
| *08 | 35 (44.87) | 33 (41.77) | 1.14 (0.61-2.13) | 0.817 | 13 (50) | 55 (41.98) | 1.38 (0.60-3.18) | 0.591 |
| *13 | 10 (12.82) | 6 (7.59) | 1.79 (0.64-5.00) | 0.413 | 3 (11.54) | 13 (9.92) | 1.18 (0.34-4.22) | 1 |
| *15 | 6 (7.69) | 12 (15. 2) | 0.46 (0.17-1.27) | 0.221 | 1 (3.85) | 17 (12.98) | 0.27 (0.04-1.67) | 0.32 |
| *18 | 3 (3.85) | 3 (3.8) | 1.01 (0.23-4.54) | 1 | 2 (7.69) | 4 (3.05) | 2.65 (0.54-13.22) | 0.571 |
| *27 | 0 (0) | 1 (1.27) | 0.00 (0.00-3.94) | 1 | 0 (0) | 1 (0.76) | 0.00 (0.00-19-78) | 1 |
| *35 | 8 (10.26) | 9 (11.39) | 0.89 (0.33-2.37) | 1 | 2 (7.69) | 15 (11.45) | 0.64 (0.16-2.72) | 0.828 |
| *37 | 3 (3.85) | 1 (1.27) | 3.12 (0.43-22.17) | 0.603 | 2 (7.69) | 2 (1.53) | 5.38 (0.90-32.07) | 0.254 |
| *38 | 1 (1.28) | 2 (2.53) | 0.50 (0.06-3.92) | 1 | 1 (3.85) | 2 (1.53) | 2.58 (0.33-20.66) | 1 |
| *39 | 1 (1.28) | 2 (2.53) | 0.5 (0.06-3.915) | 1 | 1 (3.85) | 2 (1.53) | 2.58 (0.33-20.66) | 0.996 |
| *40 | 9 (11.54) | 10 (12.66) | 0.9 (0.35-2.3) | 1 | 2 (7.69) | 17 (12.982) | 0.56 (0.14-2.34) | 0.67 |
| *41 | 2 (2.56) | 2 (2.53) | 1.01 (0.17-5.90) | 1 | 0 (0) | 4 (3.05) | 0.00 (0.00-4.90) | 0.825 |
| *44 | 7 (8.97) | 9 (11.39) | 0.77 (0.28-2.11) | 0.813 | 3 (11.54) | 13 (9.92) | 1.18 (0.34-4.22) | 1 |
| *48 | 1 (1.28) | 1 (1.27) | 1.01 (0.10-9.87) | 1 | 0 (0) | 2 (1.53) | 0.00 (0.00-9.92) | 1 |
| *50 | 16 (20.51) | 16 (20.25) | 1.02 (0.47-2.19) | 1 | 5 (19.23) | 27 (20.61) | 0.92 (0.33-2.58) | 1 |
| *51 | 13 (16.67) | 9 (11.39) | 1.56 (0.64-3.80) | 0.47 | 6 (23.08) | 16 (12.21) | 2.16 (0.78-6.02) | 0.145 |
| *52 | 9 (11.54) | 9 (11.39) | 1.01 (0.39-2.64) | 1 | 1 (3.85) | 17 (12.98) | 0.27 (0.04-1.67) | 0.318 |
| *53 | 0 (0) | 3 (3.8) | 0.00 (0.00-1.27) | 0.083 | 0 (0) | 3 (2.29) | 0.00 (0.00-6.58) | 1 |
| *56 | 1 (1.28) | 0 (0) | NA | 0.995 | 0 (0) | 1 (0.76) | 0.00 (0.00-19-78) | 1 |
| *57 | 1 (1.28) | 3 (3.8) | 0.33 (0.05-2.37) | 0.622 | 1 (3.85) | 3 (2.29) | 1.71 (0.24-12.59) | 1 |
| *58 | 14 (17.95) | 15 (18.99) | 0.93 (0.42-2.07) | 1 | 6 (23.08) | 23 (17.56) | 1.41 (0.53-3.81) | 0.7 |

Abbreviations: GF, gene frequency; CI, confidence interval; NA, not available, OR, odds ratio.

**Supplementary Table S3.** Comparative distribution of HLA-Cw alleles in 157 T1D patients with or without GADA and IA2A.

| **HLA-Cw**  **Allele** | **GADA** | | | | **IA2A** | | | |
| --- | --- | --- | --- | --- | --- | --- | --- | --- |
|  | **Positives (%GF)**  **(N=78)** | **Negatives (%GF)**  **(N=79)** | **OR (95% CI)** | **P-value** | **Positives (%GF)**  **(N=26)** | **Negatives (%GF)**  **(N=131)** | **OR (95% CI)** | **P-value** |
| *01 | 1 (1.28) | 0 (0) | Inf (0.26-inf) | 0.995 | 0 (0) | 1 (0.76) | 0.00 (0.00-19-78) | 1 |
| *02 | 4 (5.13) | 6 (7.59) | 0.66 (0.91-2.27) | 0.76 | 2 (7.69) | 8 (6.11) | 1.28 (0.29-5.74) | 1 |
| *03 | 17 (21.79) | 19 (24.05) | 0.88 (0.42-1.84) | 0.884 | 7 (26.92) | 29 (22.14) | 1.296 (0.51-3.32) | 0.783 |
| *04 | 8 (10.26) | 11 (13.92) | 0.71 (0.28-1.82) | 0.646 | 2 (7.69) | 17 (12.98) | 0.56 (0.14-2.34) | 0.67 |
| *05 | 0 (0) | 1 (1.27) | 0.00 (0.00-3.94) | 1 | 0 (0) | 1 (0.76) | 0.00 (0.00-19-78) | 1 |
| *06 | 28 (35.9) | 22 (27.85) | 1.451 (0.74-2.84) | 0.362 | 9 (34.62) | 41 (31.3) | 1.16 (0.49-2.78) | 0.919 |
| *07 | 43 (55.13) | 43 (54.43) | 1.93 (0.55-1.92) | 1 | 17 (65.38) | 69 (52.67) | 1.697 (0.72-4.01) | 0.33 |
| *08 | 2 (2.56) | 3 (3.8) | 0.67 (0.13-3.45) | 1 | 1 (3.85) | 4 (3.05) | 1.27 (0.19-8.96) | 1 |
| *12 | 10 (12.82) | 16 (20.25) | 0.58 (0.25-1.35) | 0.299 | 3 (11.54) | 23 (17.56) | 0.61 (0.18-2.09) | 0.642 |
| *14 | 4 (5.13) | 2 (2.53) | 2.08 (0.43-9.99) | 0.67 | 1 (3.85) | 5 (3.82) | 1.01 (0.15-6.92) | 1 |
| *15 | 11 (14.10) | 9 (11.39) | 1.28 (0.51-3.20) | 0.787 | 4 (15.38) | 16 (12.21) | 1.31 (0.42-4.11) | 0.904 |
| *16 | 4 (5.13) | 2 (2.53) | 2.08 (0.43-9.99) | 0.67 | 1 (3.85) | 5 (3.82) | 1.01 (0.15-6.92) | 1 |
| *18 | 1 (1.28) | 0 (0) | NA | 0.995 | 0 (0) | 1 (0.76) | 0.00 (0.00-19-78) | 1 |

Abbreviations: GF, gene frequency; CI, confidence interval; NA, not available, OR, odds ratio.
